# A Sphingosine Kinase Form 2 Knockout Sensitizes Mouse Myocardium to Ischemia/Reoxygenation Injury and Diminishes Responsiveness to Ischemic Preconditioning

**DOI:** 10.1155/2011/961059

**Published:** 2011-04-18

**Authors:** Donald A. Vessey, Luyi Li, Zhu-Qiu Jin, Michael Kelley, Norman Honbo, Jianqing Zhang, Joel S. Karliner

**Affiliations:** ^1^Liver Study Unit, Veterans Affairs Medical Center, San Francisco, CA 94121, USA; ^2^Department of Medicine, University of California, San Francisco, CA 94143, USA; ^3^Department of Pharmaceutical Science, South Dakota State University, Brookings, SD 57007, USA; ^4^Cardiology Section, Veterans Affairs Medical Center, San Francisco, CA 94121, USA; ^5^Cardiovascular Research Institute, University of California, San Francisco, CA 94143, USA

## Abstract

Sphingosine kinase (SphK) exhibits two isoforms, SphK1 and SphK2. Both forms catalyze the synthesis of sphingosine 1-phosphate (S1P), a sphingolipid involved in ischemic preconditioning (IPC). Since the ratio of SphK1 : SphK2 changes dramatically with aging, it is important to assess the role of SphK2 in IR injury and IPC. Langendorff mouse hearts were subjected to IR (30 min equilibration, 50 min global ischemia, and 40 min reperfusion). IPC consisted of 2 min of ischemia and 2 min of reperfusion for two cycles. At baseline, there were no differences in left ventricular developed pressure (LVDP), ± d*P*/d*t*max, and heart rate between SphK2 null (KO) and wild-type (WT) hearts. In KO hearts, SphK2 activity was undetectable, and SphK1 activity was unchanged compared to WT. Total SphK activity was reduced by 53%. SphK2 KO hearts subjected to IR exhibited significantly more cardiac damage (37 ± 1% infarct size) compared with WT (28 ± 1% infarct size); postischemic recovery of LVDP was lower in KO hearts. IPC exerted cardioprotection in WT hearts. The protective effect of IPC against IR was diminished in KO hearts which had much higher infarction sizes (35 ± 2%) compared to the IPC/IR group in control hearts (12 ± 1%). Western analysis revealed that KO hearts had substantial levels of phosphorylated p38 which could predispose the heart to IR injury. Thus, deletion of the SphK2 gene sensitizes the myocardium to IR injury and diminishes the protective effect of IPC.

## 1. Introduction

Exposure of the myocardium to extended periods of ischemia results in cell injury. Much of this damage occurs upon reperfusion, and thus it is referred to as ischemia/reoxygenation (I/R) injury. Reactive oxygen species are formed upon reperfusion, and they have been implicated as contributing factors to this injury [1]. I/R injury is characterized by poor recovery of hemodynamic function upon reperfusion and the development of extensive areas of infarction [2]. Protection against I/R injury can be provided by ischemic preconditioning (IPC). IPC consists of short periods of I/R that precede long term (index) ischemia and reperfusion. The triggers for IPC are cellular agonists that are released from myocytes via pannexin-1/P2X_7_ channels [3] in response to brief ischemia. These agonists bind to G-protein coupled receptors initiating a protective response [4–6]. These include adenosine, bradykinin, and opioids [4, 5] and sphingosine-1-phosphate (S1P) [6].

 S1P is an important intracellular signaling molecule that regulates diverse cellular events and has both cell growth and prosurvival effects [7–9]. These cell signaling effects are in part mediated by S1P binding to specific cell surface G-protein-coupled receptors [7–10]. In the heart, S1P is cardioprotective as revealed by studies of cultured cardiac myocytes [11, 12] as well as *ex vivo* isolated hearts [3, 6, 13]. The increased myocyte viability induced by S1P may relate to activation of a prosurvival pathway that includes PI-3K/Akt and increased bcl-2 expression followed by reduced cytochrome C release and prevention of caspase activation [9, 12].

 Sphingosine kinase (SphK) is the enzyme responsible for the formation of S1P. It exhibits two isoforms (SphK1 and SphK2) in heart [14–16]. SphK1 appears to be a protective kinase, as its overexpression increases intracellular S1P content and promotes cell growth and survival [17–19]. Indeed, study of an SphK1 null mouse demonstrated that loss of SphK1 sensitizes the myocardium to I/R injury and results in the loss of both ischemic preconditioning and ischemic postconditioning [20, 21].

 The role of SphK2 in cell survival is less clear. Studies of SphK2 in cell systems have suggested that SphK2 is antiproliferative and proapoptotic [22–24]. In contrast, it has recently been shown that SphK2-directed S1P synthesis in mitochondria is important for proper assembly of the respiratory chain [25], and thus SphK2 activity might serve to limit free radical production by the respiratory chain. In this scenario, SphK2 would actually be protective. Discerning the role of SphK2 is important particularly with respect to aging as we have found that SphK2 activity, but not SphK1, decreases with aging [26]. However, to date, no studies of the role of Sphk2 in cellular injury to heart have been reported. Thus, the primary objective of the present study was to utilize mouse hearts that possess an inactivated SphK2 gene to determine if SphK2 affects sensitivity to myocardial IR injury or cardioprotection conferred by IPC.

## 2. Results

### 2.1. Baseline Characteristics

The baseline parameters for WT and SphK2 KO mouse hearts are not different. For WT and KO, respectively, there were no differences in body weight (25.4 ± 1.2 g versus 25.9 ± 1.8 g), heart weight (93 ± 20 versus 100 ± 20 mg), heart rate (435 ± 57 versus 443 ± 59 bpm), or left ventricular developed pressure (LVDP) at baseline (106 ± 4 versus 109 ± 10 mmHg). The KO mice exhibit no evident phenotype, breed normally, have normal vascular development, and live a normal lifespan.

### 2.2. Verification of Deletion of SphK2 and Loss of SphK2 Activity

**Figure 1:**
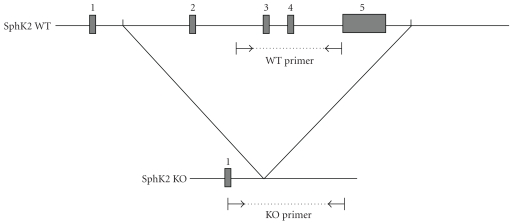
PCR Primers showing amplification of SphK2 from wild-type (+/+) and null (−/−) mice. The WT primer was used for the identification by PCR of the wild-type gene, and the KO primer was used for the detection of the null genotype. See Section 4 for details. Exons are shown as grey rectangles.

**Figure 2:**
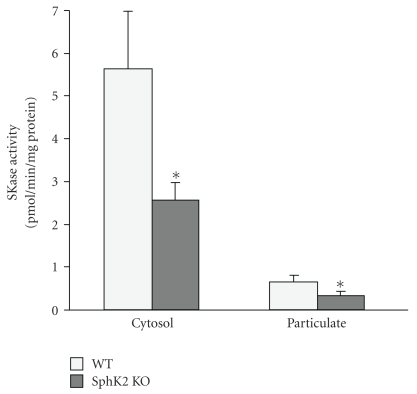
Sphingosine kinase activity (SKase) in WT and KO hearts. Hearts from WT and KO hearts were flushed free of blood and then subfractionated into cytosolic and particulate fraction as described in Section 4. The fractions were assayed for SKase activity which is expressed as pmoles per min per mg fraction protein and reveal reduced activity in the KO fractions (*P < .05 versus WT, *n* = 5).

 PCR analysis of DNA from tail snips (Figure 1) revealed that the SphK2 null hearts are indeed lacking a full length SphK2 gene. Hearts from SphK2 null mice and WT mice were subfractionated into cytosolic and particulate fractions and assayed for total sphingosine kinase activity (Figure 2). WT mice had a cytosolic specific activity of 5.63 ± 1.4 pmol/min/mg protein and a particulate fraction activity of 0.6 ± 0.2 pmol/min/mg. In contrast, KO hearts had a cytosolic specific activity of 2.65 ± 0.32 pmol/min/mg protein and a particulate fraction activity of 0.3 ± 0.08 pmol/min/mg. Total activity was reduced by approximately 53% in KO hearts relative to the wild-type hearts (53% reduction in the cytosolic fraction and 50% reduction in the particulate fractions). There was no change in the distribution of activity between cytosolic and particulate fractions with ca. 85% of total activity being present in the cytosolic fraction of both KO and WT hearts. The finding of SphK activity in the particulate fraction of KO hearts is consistent with the report of SphK activity in the mitochondria from SphK2 KO hearts [25].

**Figure 3:**
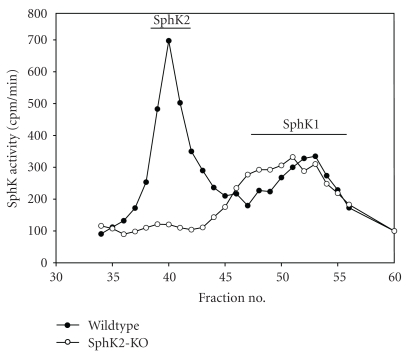
Gel filtration profile of cytosolic SKase Activity from WT and KO hearts. The cytosolic fraction isolated from pooled mouse hearts from WT (-●-) and KO (-∘-) hearts were chromatographed on a Sephacryl S200 gel filtration column. The eluted fractions were assayed for SphK activity as described in Section 4, and activity is expressed as cpm of S1P formed per min per 0.1 mL of fraction. The profile is representative of 3 separate experiments and reveal that SphK2 activity is not present in the cytosols from the SphK 2 KO hearts.

 To verify that SphK2 activity was absent in KO hearts, we took advantage of our previous finding that cytosolic SphK1 and SphK2 activity in heart can be cleanly separated by gel filtration chromatography [16]. Analysis of the cytosolic fractions from WT and SphK2 KO hearts by gel filtration analysis is shown in Figure 3. As expected, SphK2 KO hearts are completely devoid of cytosolic SphK2 activity, but SphK1 activity is not significantly altered.

### 2.3. Deletion of SphK2 Impairs Cardiac Function during Ischemia/Reoxygenation (IR) Injury

**Figure 4:**
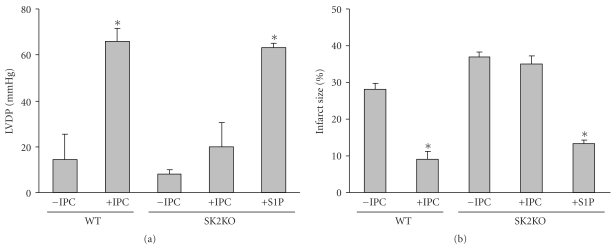
Hemodynamic function and infarct sizes in wild-type and SphK2 KO (SK2KO) hearts in ischemia-reoxygenation injury and ischemic preconditioning. Equilibrated *ex vivo* hearts were exposed to 40 min of ischemia followed by 40 min of reoxygenation either in the absence of any preconditioning (−IPC) or with ischemic preconditioning (+IPC) or with pharmacologic preconditioning using 0.4 *μ*M S1P (+S1P). Data are expressed as mean ± SEM. *P < .05 versus −IPC. *n* = 5–7/group. (a) Maximum left ventricular developed pressure (LVDP) achieved during 40 min of reperfusion expressed as % recovery relative to the preischemic value. LVDP is shown for wild-type (WT) and SphK2 knockout (SK2KO) mouse hearts. (b) Infarct size expressed as percent of the area at risk (which is the total heart area in this global ischemia model) determined at the end of 40 min of reperfusion in wild-type (WT) and SphK2 knockout (SK2KO) hearts.

Both WT and KO hearts were subjected to 50 min global ischemia and 40 minutes of reperfusion (reoxygenation). The recovery of LVDP at the end of IR is shown in Figure 4. While there was wide variation in recoveries of LVDP due to stunning, based on one way ANOVA there still was significantly lower recovery of LVDP in KO mouse hearts (8 ± 2%) than in WT hearts (22 ± 9%). Thus, IR caused more serious impairment of both cardiac hemodynamic function in KO hearts compared to the dysfunction observed in hearts from WT mice. Moreover, infarct size was significantly higher in KO hearts than in WT hearts (37 ± 1% for KO versus 28 ± 1%, in WT, *n* = 5, *P* < .05).

### 2.4. Deletion of SphK2 Prevents Cardioprotection during Ischemia/Reoxygenation Injury and Ischemic Preconditioning

As shown in Figure 4, ischemic preconditioning (IPC) increased cardiac performance in WT hearts. At the end of IR, WT-IPC hearts exhibited improved LVDP (66 ± 5% recovery) compared with the WT control group (22 ± 9%). In WT hearts infarct size was far less after IPC + IR (9 ± 2%) compared with IR alone (28 ± 1%). However, with KO hearts IPC did not provide protection. Infarct size was not diminished (35 ± 2% after IPC compared to 37 ± 1% for untreated) and was much greater than that seen with IPC-treated WT hearts (9 ± 2%). Further, recovery of LVDP was not significantly different ± IPC for KO hearts (8 ± 2% no IPC versus 20 ± 14% plus IPC) and was significantly less than LVDP obtained with IPC-treated WT hearts (66 ± 5%, *n* = 4, *P* < .05).

### 2.5. Exogenous Sphingosine-1-Phosphate Exerts Cardioprotection in SphK2 KO Hearts

 Previous results from our laboratory in [13, 20, 27] revealed that WT mouse hearts can be pharmacologically preconditioned with S1P. The data in Figure 4 indicate that pharmacological preconditioning with 0.4 *μ*M S1P protected *SphK2 *null mouse hearts against IR injury. S1P improved LVDP recovery (63 ± 2% versus 22 ± 9% untreated) and significantly reduced infarction size (13 ± 1% versus 28 ± 1% untreated). This indicates that the signaling pathway for preconditioning is still intact in KO hearts.

### 2.6. Phosphorylation Levels of Akt, Erk, and p38 and in Wild-Type and SphK2 KO Hearts

**Figure 5:**
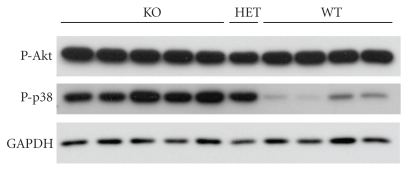
Levels of phospho-Akt (P-Akt) and phospho-p38 (P-p38) in wild-type (WT), heterozygous (HET), and SphK2-knockout (KO) mouse hearts. P-Akt and P-p38 levels were determined by Western analysis in the cytosolic fractions from 4 KO hearts (lanes 1–4), 4 WT hearts (lanes 6–9), and 1 HET heart (lane 5). GAPDH: glyceraldehyde phosphate dehydrogenase.

Phosphorylated forms of the signaling molecules Akt (P-Akt) and Erk (P-Erk) enhance survival in mouse heart. Therefore, their levels were measured by Western blotting in untreated flushed hearts from WT and KO mice. P-Akt was readily detectable in the cytosolic fraction of KO and WT hearts (Figure 5). No difference in P-Akt level was observed between WT and KO hearts (Figure 5). P-Akt was present in very low amounts in the particulate fraction from both WT and KO hearts (data not shown). P-Erk levels exhibited wide scatter, and mean values did not seem to differ between WT and KO hearts (data not shown). The detrimental signaling molecule phospho-p38 (P-p38) is present in WT mouse heart in low amount under normal conditions (Figure 5). By contrast, in KO hearts much higher levels of P-p38 were found in the cytosolic fraction (Figure 5). P-p38 protein was not readily detectable in the particulate fraction of WT or KO hearts.

## 3. Discussion

Based on studies in noncardiac cells, it has been hypothesized that SphK1 and SphK2 have opposite effects on cell survival with SphK1 being prosurvival while SphK2 is purported to be antisurvival [22–24]. We have previously shown in SphK1 KO mouse hearts that an active SphK1 is essential to cardioprotection by both ischemic preconditioning [20] and ischemic postconditioning [21]. In the current study, we have for the first time examined the role of SphK2 in cardioprotection by testing the hypothesis that elimination of SphK2 by genetic means would enhance the cardiac response to IR injury and IPC. For these studies we used a mouse model in which deletion of exons 2–5 of the SphK2 gene leads to a complete loss of SphK2 activity and thereby a >50% reduction of SphK activity in both the cytosolic and particulate fractions. A significant compensatory increase in SphK1 activity was not found. We found no significant difference in baseline cardiac function between wild-type and SphK2 null mouse hearts. However, after 40 minutes of global ischemia and 40 minutes of reperfusion, recovery of LVDP was significantly decreased in KO hearts, and infarct sizes were significantly increased. This indicates an increased sensitivity to IR injury in KO hearts. Consistent with this increased sensitivity, the capacity for IPC-induced cardiac protection was abolished in the KO hearts. In contrast to studies is isolated cells [22–24], these results provide the first evidence in a genetically modified animal that SphK2 is an important lipid kinase mediating cardiac cell survival, and further that SphK1 and SphK2 are both required for ischemic preconditioning in the heart. A protective role for SphK2 was also reported in renal IR injury [28]. However, ischemic preconditioning was not studied, and exogenous S1P receptor agonism failed to produce renal protection [28]. 

**Figure 6:**
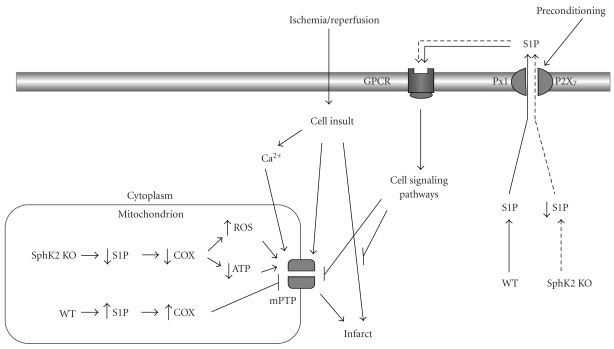
Suggested pathways for the increased susceptibility of SphK2 KO hearts to ischemia-reperfusion injury and decreased sensitivity to ischemic preconditioning. Ischemic preconditioning of wild-type (WT) hearts leads to the release of sphingosine 1-phosphate (S1P) via pannexin-1 (Px1) /P2X_7_ channels, and this S1P then binds to G-protein-coupled receptors (GPCRs) triggering cell signaling pathways that are protective in ways that include preventing the opening of the mitochondrial permeability transition pore (mPTP), which is known to trigger cell injury. In the Sphk2 KO hearts, the level of S1P is reduced below the threshold necessary to trigger protective cell signaling pathways. The mPTP can also be opened by increased mitochondrial accumulation of Ca^2+^, and this can be enhanced by reactive oxygen species (ROS). In SphK2 KO hearts, the absence of adequate levels of S1P leads to defective cytochrome oxidase (COX) assembly which could lead to the generation of ROS that likely supports mPTP opening.

 IPC results in release of S1P from myocytes which then binds to cell surface G-protein-coupled receptors and triggers the cardioprotective response [3, 6, 29]. It follows that deletion of SphK2 might make less S1P available for release during brief episodes of IPC. We have shown that a decrease in S1P response makes a significant contribution to overall potency of the preconditioning response [6]. Further, our previous work revealed that during ischemia/reperfusion injury in isolated hearts, SphK activity declines markedly during ischemia and remains depressed during recovery, while in hearts that have been preconditioned, recovery of enzyme activity is much more robust [30]. S1P levels are altered in parallel [30], and there may be a threshold concentration at the cellular level below which prosurvival pathways are either not activated or are suppressed. Thus, the simplest explanation for the failure of SphK2 KO mice to precondition is that there is insufficient intracellular S1P to achieve threshold levels of S1P release for triggering IPC (see Figure 6).

 In addition, interactions of SphK2 and/or S1P with other signaling molecules may also contribute to the loss of cardioprotection. In particular, Strub et al. have shown that mitochondrial SphK2 activity is critical for the proper assembly of cytochrome oxidase [25]. SphK2 KO mice contain a defective cytochrome oxidase that limits respiration. Decreased cytochrome oxidase activity can cause accumulation of electrons upstream, and this can lead to increased ROS generation [31]. Opening of the mitochondrial permeability transition pore (mPTP) is thought to be the key to reperfusion injury, and ROS generation encourages mPTP opening [32]. Therefore, increased ROS generation upon reoxygenation by SphK2 KO hearts could promote mPTP opening and increase subsequent injury. This would provide a link between loss of SphK2 and IR injury (see Figure 6).

 These findings have important implications with regard to the aging heart. As shown in Figure 3 and demonstrated previously [16, 20, 26], in young rodent hearts, SphK2 activity exceeds that of SphK1, but as animals age this ratio changes so that senescent rat hearts contain significantly more SphK1 than SphK2 activity [26]. The data in the present paper indicate that this shift from SphK2 to SphK1 is unlikely to affect cardioprotection per se. However, this shift does appear to lead to somewhat reduced S1P levels with aging [26], and this may contribute to the impaired response to certain cardioprotective interventions such as IPC that have been noted in aged hearts [26, 33, 34]. 

 Activation of Akt by phosphorylation plays an important role in promoting cardiomyocyte survival. Akt phosphorylation is cardioprotective both *in vitro *and *in vivo* [35, 36]. In this study, we found that the phosphorylation levels of Akt (and also Erk) were the same in SphK2-KO mouse hearts at baseline as in WT. However, the detrimental signaling molecule phospho-p38 was readily detectable in KO hearts at baseline, but low in WT hearts. This finding implies that chronic activation of p38 is present in the KO mouse. This observation is novel as it has been shown that preventing p38 phosphorylation is an important component of cardioprotection [37]. Chronic activation of p38 represents a negative predisposition of the KO hearts to injury. This could contribute to the adverse response of SphK2 KO hearts to IR and IPC.

 In summary, our data demonstrate for the first time that myocardial damage is enhanced after ischemia/reperfusion in mice null for SphK2 and that the cardioprotective intervention of preconditioning is abolished by deletion in the SphK2 gene. These observations are contrary to prior suggestions derived from *in vitro* models that SphK1 and SphK2 drive opposing functions that regulate cell fate.

## 4. Materials and Methods

This study was conducted in accordance with the *Guide for the Care and Use of Laboratory Animals *(National Academic Press, Washington DC, 1996), and all procedures were approved by the Animal Care Subcommittee of the San Francisco Department of Veterans Affairs Medical Center. Aprotinin, leupeptin, pepstatin A, and triphenyltetrazolium chloride (TTC) were obtained from Sigma. D-Erythro-sphingosine 1-phosphate (S1P) was obtained from Biomol Research Laboratories. D-Erythro-sphingosine-[^3^H] was obtained from American Radiolabeled Chemicals.

### 4.1. SphK2 Null Mice

 SphK2 null (KO) mice in which exons 2–5 of the SphK2 gene had been deleted [38] were obtained from Drs. Shaun Coughlin and Rajita Pappu (Cardiovascular Research Institute, University of California, San Francisco). These mice along with their wild-type littermates were used for all studies reported herein and were all 3 to 4 months of age at the time of study. Male homozygous null (SphK2^−/−^) and wild-type (WT) mice were generated by breeding heterozygous (SphK2^+/−^) mice. Genotyping using PCR to confirm the absence of exons 2–5 of SphK2 DNA was routinely performed on tail biopsies of 3-4-week-old mice. Figure 1 shows PCR primers used for analysis of SphK2^+/+^, SphK2^+/−^, Sphk2^−/−^ mice. To identify WT and KO mice, PCR was employed using the following primer sets. For the WT, the set was 5′ATTTTCTGGAGGGCGGGATAGG3′ and 5′AAGAGGAACGGGGAGTGAGACAAG3′, which amplifies a 572-bp fragment. The homozygous null (SphK2^−/−^) mice were identified with the primer set as follows: 5′GCCACCACTTATGAGGAGAATCG3′ and 5′GACACAGAACATCCCATCCCTAAC3′. This set amplifies a fragment of approximately 378 bp.

### 4.2. Langendorff Isolated Perfused Heart Preparation

 Male mice (3-4 months of age, weighing 25–28 g) were heparinized (500 U/kg, IP) and anesthetized with sodium pentobarbital (60 mg/kg, IP). Hearts were rapidly excised, washed in ice-cold arresting solution (NaCl 120 mmol/L, KCl 30 mmol/L), and cannulated via the aorta on a 20-gauge stainless steel blunt needle. Hearts were perfused at 70 mmHg on a modified Langendorff apparatus using Krebs-Henseleit solution containing (mmol/L) NaCl 118, KCl 4.7, CaCl_2_ 2.5, MgSO_4_ 1.2, KH_2_PO_4_ 1.2, NaHCO_3_ 24, glucose 5.5, and Na pyruvate 5.0. The perfusion solution was bubbled with 95% O_2_/5% CO_2_ and maintained at 37°C. During periods of global ischemia the hearts were lowered into a thermostated chamber to maintain a heart temperature of 37°C.

### 4.3. Ischemia-Reoxygenation (IR) and Ischemic Preconditioning (IPC) Protocols

 The protocol for IR experiments consisted of a 20-minute equilibration period, followed by 40 minutes of global ischemia and 40 minutes of reperfusion (reoxygenation). For IPC, hearts were equilibrated for 16 minutes and then subjected to two short cycles of preconditioning, each consisting of 2 minutes of global ischemia and 2 minutes of reperfusion. This was followed immediately by 50 min of global ischemia and 40 min of reperfusion. Hemodynamics (left ventricular developed pressure (LVDP), LV end-diastolic pressure (LVEDP), and ±d*P*/d*t*) were recorded using a balloon inserted into the LV as previously described [20]. LVEDP was initially set at 5 mmHg during equilibration and subsequent changes measured. Infarct size was measured by TTC staining as previously described [20].

### 4.4. Sphingosine Kinase Activity

 Hearts were rapidly excised from anesthetized mice (pentobarbital sodium 60 mg/mL ip), mounted on the Langendorff apparatus, and flushed for 1 min with perfusion buffer. They were then homogenized in ice-cold isolation buffer (0.13 M KCl, 20 mM Hepes, pH 7.4, 1 mM EGTA, 1 *μ*g/L leupeptin, 0.25 *μ*g/L each of chymostatin and pepstatin A). The homogenate was centrifuged for 6 min at 350 g in a TOMY microcentrifuge to remove cell debris and nuclei. The supernatant was centrifuged for 50 min at 100,000 g. The supernatant was decanted and designated as the cytosolic fraction. The particulate fraction was washed once with buffer A and then repelleted by centrifugation. The wash was added back to the cytosolic fraction. Both cytosolic and membrane fractions were used separately for assay of SphK activity. The radioassay uses a chloroform (fresh)/methanol/aqueous trisodium EDTA extraction system to separate reactant ([3H]sphingosine) from product ([3H]S1P) essentially as previously described [30, 39]. A standard assay contains Triton X-100 (0.05%), 250 mM KCl, 1 *μ*M [3H]sphingosine (300–400 cpm/pmol), 5 mM ATP, 10 mM Mg^2+^, 100 mM Tris, pH 8.0, 10 mM ascorbate, and enzyme protein in a volume of 0.5 mL. Separation and identification of enzymatically active forms of SphK1 and SphK2 were done as described previously [16].

### 4.5. Western Analysis

 Mouse hearts were mounted on the Langendorff apparatus and perfused under the indicated conditions. They were then homogenized in 0.13 M KCl, 20 mM HEPES pH 7.4, 1 mM EGTA, 1 *μ*g/L leupeptin, 0.25 *μ*g/L each of aprotinin and pepstatin A and centrifuged at 100,000×g to generate a particulate and a cytosolic fraction. The particulate fraction was washed once with the isolation buffer. Western analysis was conducted as described previously [26] using the following antibodies from Cell Signaling Technology: phospho-Akt (Ser 473, no. 9271), phospho-p38 (Thr180/Tyr182, no. 9211), p38 (no. 9218), phospho-Erk1/2 (Thr202/Tyr204. no. 4376). Protein concentration was determined using the detergent compatible DC Protein Assay kit from Bio-Rad and used to equalize the protein concentration in all samples. Each lane was then loaded with exactly 10 *μ*g of protein.

### 4.6. Statistical Analysis

The data are presented as mean ± SEM. The significance of the differences in mean values for hemodynamics, infarction size, between groups was evaluated by one-way ANOVA, followed by posthoc testing (Newman-Keuls). Differences in SphK activity between WT and SphK2 KO tissues were evaluated by Student's *t*-test. *P* < .05 was considered statistically significant.
